# Phase I study of everolimus and mitomycin C for patients with metastatic esophagogastric adenocarcinoma

**DOI:** 10.1002/cam4.77

**Published:** 2013-04-02

**Authors:** Dominique Werner, Akin Atmaca, Claudia Pauligk, Anette Pustowka, Elke Jäger, Salah-Eddin Al-Batran

**Affiliations:** 1 Krankenhaus Nordwest, UCT-University Cancer CenterFrankfurt am Main, Germany; 2 Novartis Pharma GmbHNürnberg, Germany

**Keywords:** Esophagogastric, everolimus, gastric, mitomycin C, RAD001

## Abstract

This study aimed at determining the recommended dose of the mammalian target of rapamycin inhibitor everolimus in combination with mitomycin C (MMC) in patients with previously treated metastatic esophagogastric cancer. In this phase I trial, patients received escalated doses of oral everolimus (5, 7.5, and 10 mg/day) in combination with intravenous MMC 5 mg/m^2^ every 3 weeks. Endpoints were the dose-limiting toxicity (DLT), safety, and response rates. Tumor tissues were tested for HER2-status and mutations in the *PTEN, PIK3CA*, *AKT1*, *CTNNB1,* and E-cadherin type 1 genes. Sixteen patients (12 male, four female) with gastric/gastroesophageal junction cancer were included. All patients were previously treated with a platinum-based chemotherapy. Treatment cohorts were: 5 mg/day, three patients; 7.5 mg/day, three patients; and 10 mg/day, 10 patients. No DLTs occurred during dose escalation. Most frequent grade 3 toxicities were leukopenia (18.8%) and neutropenia (18.8%). All other grade 3 toxicities were below 10%. No grade 4 toxicities occurred. Three (18.8%) patients experienced partial responses and four patients had stable disease (SD). Antitumor activity according to Response Evaluation Criteria In Solid Tumors (RECIST)-criteria was highest in the 10 mg/day cohort. No associations between HER2-status or detected mutations and response were observed. The recommended dose of everolimus combined with MMC is 10 mg/day. Encouraging signs of antitumor activity were seen (http://www.ClinicalTrials.gov; Clinical trial registration number: NCT01042782).

## Introduction

Gastric cancer is often diagnosed in locally advanced or metastatic stages and, therefore, of poor prognosis. Systemic chemotherapy is widely accepted as palliative treatment, leading to objective responses, improvement of the quality of life, and prolonged survival [1–3]. Based on response results of several combination chemotherapy regimens, advanced gastric cancer is considered to be a chemotherapy-sensitive disease. However, results of survival have been unsatisfactory so far, with a median survival time ranging between 6 and 8 months [4].

The therapeutic standard in the first-line setting for gastric cancer or cancer of the esophagogastric junction (EGJ) is either cisplatin/5-FU, oxaliplatin/5-FU, with or without epirubicin or docetaxel. Capecitabine could replace 5-FU in most of the schedules. At the time this study was designed, there was no chemotherapy regimen considered to be the standard of care in the second line for patients with advanced gastric cancer. Most recently, taxanes and irinotecan have been proved effective in the second-line setting [5]. However, new protocols remain warranted in this setting.

Everolimus is a derivative of rapamycin and has been investigated as an anticancer agent based on its potential to act directly on the tumor cells by inhibiting tumor cell proliferation and tumor growth in situ. The target of everolimus is mTOR (mammalian target of rapamycin), a serine–threonine kinase which is a member of the larger PI3K (phosphatidylinositol 3-kinase) family and present in all cells. Several preclinical studies have indicated dysregulation of mTOR activity in gastric cancer cell models. Mutations in upstream regulators of mTOR signaling pathway epithelial growth factor receptor [6] (EGFR), PI3K [6], and PTEN [7] have been observed in patient-derived gastric tumor samples. Patient-derived gastric cancer samples have been shown to express phosphorylated mTOR indicative of mTOR activation [8–11], which has been positively correlated with tumor progression and poor survival in patients with gastric cancer [9, 11, 12]. mTOR inhibitors alone or in combination with other agents significantly delayed tumor progression in xenograft models of gastric cancer [8, 13].

Mitomycin C (MMC) represents a well-tolerable, active, and low-cost chemotherapy. As a single agent, MMC at 10–15 mg every 6–8 weeks is considered one of the most active single agents in gastric cancer, as it has achieved consistent response rates in the 20–30% range [14]. It is approved for gastric cancer in all settings and is accepted as an option for patients with gastric cancer who failed first-line treatment. Previous studies have already established the efficacy of MMC in the treatment of gastric cancer and have shown that it is well tolerable [15]. The toxicity profile of MMC is mainly hematological. Dose-limiting toxicities (DLTs) are leukopenia and thrombocytopenia. When this study was designed, the efficacy of new drugs in the second-line treatment of gastric cancer such as irinotecan and paclitaxel was unknown. Furthermore, everolimus monotherapy was under evaluation in phase I trials.

In this study, we conducted a phase I trial of everolimus in combination with MMC to determine the recommended dose and the DLT of everolimus plus MMC in advanced gastric cancer or cancer of the EGJ for a future randomized trial.

## Materials and Methods

### Patient eligibility

Main eligibility criteria were patients ≥18 years of age with histologically confirmed diagnosis of metastatic gastric cancer or adenocarcinoma of the EGJ; CT or MRI scan had to demonstrate measurable disease by Response Evaluation Criteria In Solid Tumors (RECIST)-criteria; and at least one prior chemotherapy in the palliative setting or progressive disease under adjuvant or neoadjuvant therapy within 6 months of treatment start date. Further criteria were Eastern Cooperative Oncology Group performance status (ECOG) ≤1, Life expectancy >4 months, sufficient renal, hepatic and bone marrow function.

Participants gave written informed consent before they entered the study, which was approved by the responsible ethics committee.

### Treatment

Patients received MMC 5 mg/m^2^ intravenously as a bolus injection every 3 weeks in combination with oral doses of everolimus at 5 mg/7.5 mg/10 mg per day starting 3 days prior to MMC. Cycles were repeated every 3 weeks. A 3-patient cohort, dose-escalating study design was used. The initial dose of everolimus was 5 mg and was increased by 2.5 mg in the next cohort, provided that all patients in the previous cohort finished their first cycle of treatment without experiencing a DLT. In case a patient experienced a DLT, three additional patients were enrolled at the same dose level. If only one of the six patients treated at the same dose level experienced a DLT, the trial continued at the next higher dose level. If two or more patients out of the six exhibited DLT, the maximum tolerated dose (MTD) was supposed to be surpassed and dose escalation stops at that level. Intrapatient dose escalation was not permitted. It was predefined that dose escalation of everolimus will be stopped at 10 mg/day in case a MTD is not achieved. Patients were treated with everolimus until progression of tumor, the occurrence of unacceptable toxicity, or until the investigator or patient decided that continuation is not in the best interest of the patient.

### Toxicity evaluation and dose adjustments

Toxicity was assessed using the National Cancer Institute Common Terminology Criteria for Adverse Events (NCI-CTCAE), version 3.0. DLT was defined as a hypersensitivity reaction ≥grade 2, any ≥grade 3 nonhematologic toxicity (except alopecia and mucositis lasting less than 7 days), grade 4 thrombocytopenia and grade 4 neutropenia persisting >5 days. Adverse events were considered dose limiting when they were at least possibly related to the study treatment, that is, the combination of everolimus and MMC, and they were considered for the definition of the MTD when they occurred during the first cycle of treatment (first cycle DLT). Abnormal laboratory values were considered dose limiting when they, in addition, were considered as clinically significant. For patients who were unable to tolerate the protocol-specified everolimus dosing schedule, the dose of everolimus was adjusted in 2.5 mg reduction steps.

### Response and survival evaluation

Objective responses were defined based on RECIST-criteria (version 1.0). Progression-free survival (PFS) was defined as the time from first study drug administration to objective tumor progression or death from any cause. Overall survival (OS) was defined as the time from first study drug administration to death from any cause. The interval of tumor assessment in response was 6 weeks during the treatment and 3 months after end of study. Survival evaluation was also 3 months after end of study.

### Biomarker studies

Patient tissue was analyzed for hot spot mutations or polymorphisms in phosphatase and tensin homolog (*PTEN)*, phosphatidylinositol-4,5-bisphosphate 3-kinase, catalytic subunit alpha (*PIK3CA*), v-akt murine thymoma viral oncogene homolog 1 (*AKT1*), cadherin-associated protein beta 1 (*CTNNB1*), and E-cadherin type 1 (*CDH1*) with conventional DNA sequencing (ABI 3500DX Genetic Analyzer, Applied Biosystems, Foster City, CA) and optimized standard PCR conditions. DNA was extracted from paraffin-embedded tumor tissue using QIAamp DNA Tissue Kit (Qiagen, Hilden, Germany).

The primer sequences were *PTEN* (exon 2) F: 5′-TGACCACCTTTTATTACTCC-3′, R: 5′-AGTATCTTTTTCT GTGGC-3′, *PTEN* (exon 3) F: 5′-CTACTC TAAACCCATAGAAGG-3′, R: 5′-CCTCACTCTAACAAGCAG-3′, *PTEN* (exon 5) F: 5′-GCAACATTTCTAAA-GTTACCTAC-3′, R: 5′-CAATAAATTCTCAGATCCAGG-3′, *PTEN* (exon 6) F: 5′- CAT-AGCAATTTAGTGAAATAACT-3′, R: 5′- GATATGGT TAAGAAAACTGTTC-3′, *PTEN* (exon 7) F: 5′- TGACAGTTTGACAGTTAAAGG-3′, *PTEN* (exon 8) F: 5′-GCAACATTT-CTAAAGTTACCTAC-3′, R: 5′-CATACATACAAGTCAACAACC-3′, *PTEN* (exon 9) F: 5′-GAGTCATATTTGTGGGTT-3′, R: 5′-GACACAATGTCCTA TTGCCAT-3′, *Akt1* (exon 4) F: 5′-CACACCCAGTT CCTGCCT-3′, *CTNNB1* (exon 3) F: 5′- GCTGATTTGATGGA-GTTGGA-3′, R: 5′-GCTACTTGTTCTTGAGTGAA-3′, *CDH1* (exon 6) F: 5′-CTC-ACTTGGTTCTTTCAG-3′, R: 5′-AACCTTTGGGCTTGGACA-3′, *CDH1* (exon 7) F: 5′-AGCTTGTCTAAACCTTCATC-3′, R: 5′-GCTTAGACCA TCACTGTATT-3′, *PIK3CA* (exons 10, formerly exons 9) F: 5′- GATTGGTTCTTTCCTGTCTCTG-3′, R: 5′- CCACAA-ATATCAATTTACAACCATTG-3′, and *PIK3CA* (exon 21, formerly exon 20) F: 5′-CATTTGCTCCAAACTGACCA-3′, R: 5′-TGTGGAATCCAGAGTGAGCTT-3′.

To determine the HER2-status, immunohistochemistry (IHC; 4B5 antibody) and silver in situ hybridization (SISH) were used. The IHC and SISH results were interpreted using the scoring scheme proposed for gastric cancer by Hofmann et al. [16] (ToGA score) and Rüschoff et al. [17].

## Results

### Patients

Sixteen patients were enrolled in three treatment cohorts at three dose levels: 5 mg/day, three patients; 7.5 mg/day, three patients; and 10 mg/day, 10 patients (the 10 mg cohort was extended to 10 patients as an MTD was not achieved and no further escalation was planned). Patients' characteristics are summarized in Table 1. Twelve patients were male and the median age was 63 (range, 36–88) years. All patients were pretreated with a median of 2 (range, 1–6) prior chemotherapy lines. All patients had received a platinum-based chemotherapy and almost all patients (14/16) had also received docetaxel.

**Table 1:** Patients characteristics

Patients characteristics	No. of patients (%)*n* = 16
Sex	
Male	12 (75.0)
Female	4 (25.0)
Age	
Median age, years (range)	63 (36–88)
ECOG performance status	
0	5 (31.3)
1	11 (68.8)
Primary tumor location	
Gastroesophageal junction	6 (37.5)
Mid to distal stomach	10 (62.5)
No. of organs involved (primary tumor excluded)	
1	3 (6.3)
2	5 (31.3)
3	3 (6.3)
4	5 (31.3)
Organs involved (primary tumor excluded)	
Liver	12 (75.5)
Lymph nodes	10 (62.5)
Peritoneum	7 (43.8)
Lung	4 (25.0)
Other^1^	9 (56.3)
Lauren classification	
Diffuse/mixed	6 (37.5)
Intestinal	10 (62.5)
No. of previous lines of chemotherapy	
1	5 (31.3)
2	6 (37.5)
≥3	5 (31.3)

ECOG, Eastern Cooperative Oncology Group.

1 Other: adrenal gland, spleen, pancreas, bones, duodenum, rectum, adrenal, retroperitoneal lymphoma.

### Safety and DLT

Median treatment duration was 52 days (range, 13–321 days). A total of 51 cycles of chemotherapy/everolimus were administered with an overall median of 2 cycles (range, 1–12). Median numbers of cycles administered per cohort were 2 (range, 1–4) in the 5 mg cohort, 2 (range, 2–2) in the 7.5 mg cohort, and 3 (range, 1–12) in the 10 mg cohort. Adverse events according to cohort and in the total population are shown in Table 2. The most commonly observed all grade toxicities, possibly related to the treatment were leukopenia in 10 patients (62.5%), nausea in 10 patients (62.5%), neutropenia in nine patients (56.3%), mucositis/stomatitis in nine patients (56.3%), alopecia in eight patients (50.0%), and thrombocytopenia in eight patients (50.0%). The most commonly observed grade 3 toxicities were leukopenia in three patients (18.8%) and neutropenia in three patients (18.8%). The most commonly observed grade 3 toxicities were leukopenia (18.8%) and neutropenia (18.8%). Leukopenia and neutropenia were the only grade 3 events in the 5 mg cohort, observed in one patient each. No grade 3 toxicities were documented in the 7.5 mg cohort. In the 10 mg cohort, several grade 3 events were observed, which comprised leukopenia and neutropenia in two patients each, and mucositis, lymphopenia, anemia, and diarrhea with infection in one patient each.

**Table 2:** Side effects with possible relationship to everolimus/mitomycin C

	5.0 mg (*n* = 3)*n*			7.5 mg (*n* = 3)*n*			10.0 mg (*n* = 10)*n*			Total pts for all cohorts (*n* = 16)*n*		
				
Adverse event	G1/G2	G3	Total	G1/G2	G3	Total	G1/G2	G3	Total	G1/G2	G3	Total
Leukopenia	1	1	2	1	–	1	5	2	7	7	3	10
Nausea	1	–	1	3	–	3	6	–	6	10	–	10
Oral mucositis/stomatitis	1	–	1	3	–	3	4	1	5	8	1	9
Neutropenia	1	1	2	1	–	1	4	2	6	6	3	9
Alopecia	1	–	1	–	–	–	7	–	7	8	–	8
Thrombocytopenia	1	–	1	–	–	–	7	–	7	8	–	8
Diarrhea	1	–	1	1	–	1	2	1	3	4	1	5
Lymphopenia	–	–	–	–	–	–	4	1	5	4	1	5
Anemia	3	–	3	1	–	1	–	1	1	4	1	5
Fatigue	1	–	1	–	–	–	4	–	4	5	–	5
Dry skin, rash or desquamation	–	–	–	–	–	–	4	–	4	4	–	4
Hyperglycemia	–	–	–	–	–	–	3	–	3	3	–	3
Vomiting	–	–	–	1	–	1	2	–	2	3	–	3
Peripheral neuropathy	1	–	1	–	–	–	2	–	2	3	–	3
Infection	–	–	–	–	–	–	1	1	2	1	1	2
Fever	1	–	1	1	–	1	–	–	–	2	–	2
Hypoalbuminemia	1	–	1	1	–	1	–	–	–	2	–	2
Hyperbilirubinemia	–	–	–	–	–	–	1	1	2	1	–	2
Pain	–	–	–	–	–	–	1	–	1	1	–	1
Pruritus/itching	–	–	–	–	–	–	1	–	1	1	–	1
Edema: limb	–	–	–	1	–	1	–	–	–	1	–	1
Constipation	–	–	–	1	–	1	–	–	–	1	–	1
Hypocalcemia	1	–	1	–	–	–	–	–	–	1	–	1
Dizziness	1	–	1	–	–	–	––	–	–	1	–	1
GGT elevation	1	–	1	–	–	–	–	–	–	1	–	1
Insomnia	1	–	1	–	–	–	–	–	–	1	–	1
Hypertriglyceridemia	1	–	1	–	–	–	–	–	–	1	–	1

GGT, gamma-glutamyl transpeptidase.

For dose escalation, first cycle DLTs were considered. No DLTs during the first cycle were observed in any of the cohorts during the dose escalation phase. No DLTs were observed in the 5 and 7.5 mg in any cycle. Also no DLTs occurred in the first three patients of the 10 mg cohort. As a further dose escalation was not planned and to gain more information on safety and tolerability, the 10 mg cohort was extended to a total of 10 patients. In the extended phase, one DLT was observed in patient #14. The patient suffered grade 3 diarrhea accompanied by bacterial infection and dehydration during the second cycle of his treatment. A causal relation to the study treatment could not be excluded.

### Activity

**Figure 1:**
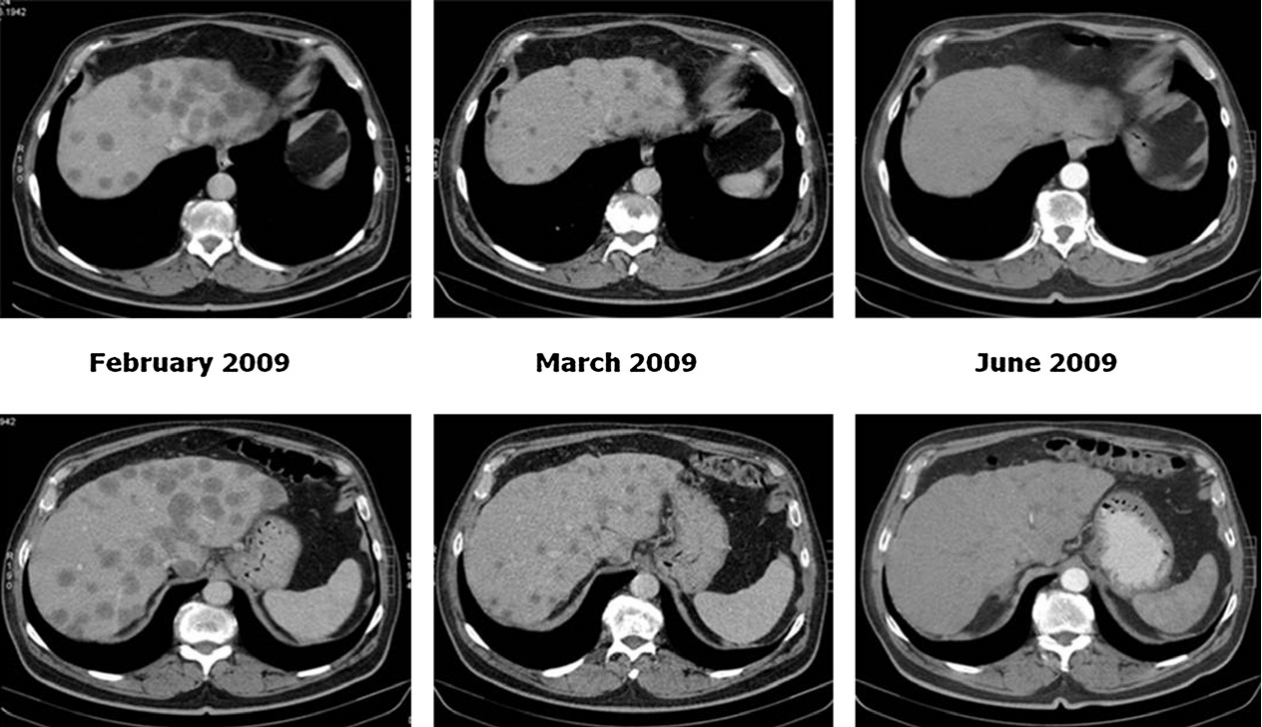
Objective response under everolimus/MMC treatment in patient #9.

The characteristics of disease together with important clinico-pathological criteria are given in Table 3. On first response evaluation, there were three patients (#2, #9, and #14) with documented partial response, one patient (#2) in the 5 mg cohort and two patients (#9 and #14) in the 10 mg cohort. Patient #9 in the 10 mg cohort had a strong reduction of multiple liver metastases, lasting more than 11 months (Fig. 1). Response rates favored the 10 mg cohort, in which four patients had SD and one patient had a long lasting objective response. Additionally the rates of progressive disease as best response were 2/3 (66.6%), 3/3 (100%), and 3/10 (30%) in the 5, 7.5, and 10 mg cohorts, respectively. Median OS for patients in the 5, 7.5, and 10 mg cohorts were 2.6, 3.5, and 7.2 months, respectively, and median PFS were 1.5, 1.6, and 2.6 months, respectively.

**Table 3:** Characteristics of disease and selected clinico-pathological baseline criteria

No.	Tumor location	Lauren classification	ECOG	Prior therapy	Organs involved	HER2-status^1^	Genetic variants	Dose cohort	No. of Cycles	Best response	Response/SD duration [months]
**1**	Stomach	Intestinal	1	FLO	LN	IHC 3+	Wild type	5 mg	1	PD	–
**2**	Stomach	Diffuse	1	FLOT	LN, peritoneal, liver	IHC 1+	Wild type	5 mg	4	PR	3.1
**3**	GEJ	Intestinal	1	DCF; FOLFIRI; FOLFIRI/cetuximab;FLOT; sunitinib; capecitabine	LN, liver, lung, other	IHC 1+	PTEN Ex2 p.G36E	5 mg	2	PD	–
**4**	Stomach	Diffuse	1	FLOT	Peritoneal, liver, other	IHC 0	PTEN Ex2 SNP rs1903858;PTEN Ex9 p.H397Y	7.5 mg	2	PD	–
**5**	Stomach	Diffuse	1	ECX, FLOT	LN, other	IHC 1+	Wild type	7.5 mg	2	PD	–
**6**	Stomach	Diffuse	1	FLO	LN, peritoneal, liver	IHC 0	Wild type	7.5 mg	2	PD	–
**7**	Stomach	Intestinal	1	PLF; docetaxel	LN, liver	IHC 0	Wild type	10 mg	2	PD	–
**8**	Stomach	Intestinal	1	FLOT; CF; FLO	Peritoneal, liver, other	IHC 3+	AKT1 SNP rs3730358;PTEN Ex2 SNP rs1903858	10 mg	2	PD	–
**9**	GEJ	Diffuse	0	FLOT	Liver, other	IHC 0	Wild type	10 mg	12	PR	11
**10**	GEJ	Diffuse	0	FLOT	LN, liver	IHC 0	PTEN Ex2 SNP rs1903858	10 mg	1	NE	–
**11**	Stomach	Intestinal	1	FLOT; FLO	LN	IHC 0	Wild type	10 mg	3	SD	2.5
**12**	Stomach	Intestinal	1	FLOT; FLO	Peritoneal, liver, lung, other	IHC 2 + /SISH 2.0	Wild type	10 mg	3	SD	2.8
**13**	GEJ	Intestinal	1	FLO; FOLFIRI	Liver, lung	NA	PTEN Ex2 SNP rs1903858	10 mg	4	SD	3
**14**	Stomach	Intestinal	0	ECF; PLF; DCF; capecitabine;XELIRI; XP	LN, peritoneal	IHC 0	Wild type	10 mg	2	PR	3.3
**15**	GEJ	Intestinal	0	CF; FLOT; FOLFIRI	Liver	IHC 0	Wild type	10 mg	2	PD	–
**16**	GEJ	Intestinal	0	PLF; FOLFIRI; FLOT	LN, peritoneal, liver, lung	IHC 2 + /SISH 0.9	Wild type	10 mg	7	SD	5

PR, partial response; SD, stable disease; PD, progressive disease; NA, not assessed; HER2, human epidermal growth factor receptor 2; IHC, immunohistochemistry; SISH, silver in situ Hybridization; Chr17, chromosome 17; SNP, single nucleotide polymorphism; Ex, exon; GEJ, gastroesophageal junction; LN, Lymph nodes; FLO, 5-FU, leucovorin, oxaliplatin; FLOT, 5-FU, leucovorin, oxaliplatin, docetaxel; DCF, docetaxel, cisplatin, 5-FU; FOLFIRI, leucovorin, 5-FU, irinotecan; ECX, cisplatin, epirubicin, capecitabine; PLF, cisplatin, leucovorin, 5-FU; CF, cisplatin, 5-FU; ECF, epirubicin, cisplatin, 5-FU; DCF, docetaxel, cisplatin, 5-FU; XP, capecitabine, cisplatin; ECOG, Eastern Cooperative Oncology Group.

1 **HER2 positivity** defined as: IHC 3+ or 2+ and SISH ≥2.0 HER2:Chr17.

### Correlative studies

Three of 15 (20%) assessed patients showed HER2 overexpression (Table 3). In the mutational analysis, a total of three genetic variants were identified in the region of *PTEN* and one in the *AKT1* gene (Table 3). Two of the variants, single nucleotide polymorphism (SNP) rs1903858 in *PTEN* and SNP rs3730358 in *AKT1*, were intronic SNPs and two were missense substitutions (p.H397Y and p.G36E, both in *PTEN*). All four detected variants had already been previously described (http://www.ncbi.nlm.nih.gov/SNP/; http://www.sanger.ac.uk/genetics/CGP/cosmic/). *PIK3CA*, *AKT1*, *CTNNB1,* or *CDH1* hotspot mutations were not found. None of the genetic variants evaluated was observed in patient number nine who had a favorable clinical development.

## Discussion

We performed a dose escalation Phase I study of everolimus in combination with the cytotoxic agent MMC in patients with advanced gastric cancer or cancer of the EGJ who were resistant to prior standard chemotherapy. We demonstrated that oral everolimus at the standard dose of 10 mg/day can be safely combined with a cytotoxic drug such as MMC. The adverse events observed were generally mild. With the exception of hematological toxicity, which was most likely related to MMC (leukopenia 18.8%, neutropenia 18.8%, and anemia 6.3% for all doses), individual grade 3 adverse events possibly related to the treatment did not exceed the 10% range in any cohort. Unexpected toxicities and grade 4 toxicities did not occur and the overall side effects were consistent with previous reports, in which frequently occurring adverse events related to everolimus were stomatitis/oral mucositis, fatigue, anorexia, hyperglycemia, hyperlipidemia, elevated liver enzymes, diarrhea, and hypophosphatemia [18–21]. Most grade 3 toxicities in our trial were seen in the 10 mg cohort. This may be related to the higher dose, but also to longer study drug exposure in this cohort, where individual patients were treated up to 11 months and were more likely to experience toxicities. One important finding in our trial is that everolimus could be combined with the cytotoxic agent MMC at the standard 10 mg per day dose, which is the recommended dose for everolimus in the monotherapy setting. This is in line with a recent study, which evaluated everolimus in combination with paclitaxel and trastuzumab for patients with breast cancer and HER2 overexpression. In this phase Ib dose escalation study, 30 patients were treated with everolimus at different doses in combination with paclitaxel and trastuzumab. Grade 3 to 4 neutropenia was the most common toxicity (52%) and everolimus at 10 mg/day was recommended for additional development [22]. Since we chose the first cycle DLT (3 weeks) as the primary endpoint, the long-term tolerability should be reevaluated carefully in future phase II trials, if MMC is combined with everolimus. This is particularly important because MMC is known to cause prolonged myelosuppression.

Some signs of antitumor activity were seen. There were three patients with objective responses, one of whom had a dramatic and durable response, lasting 11 months. The antitumor activity according to RECIST-criteria seemed to be dose dependent, as all disease stabilizations and two of three partial responses (of which one was durable) occurred in the 10 mg cohort. Treatment duration and survival time were also longer in the 10 mg cohort than they were in the lower dose cohorts. This makes it more likely that everolimus contributed to the activity observed in some patients and is in line with encouraging signs of antitumor activity that have been observed in early trials with everolimus as single agent. In a phase I study of everolimus in nine Japanese patients with advanced solid tumors, everolimus 10 mg/day resulted in a partial response with a duration of >4 months in a heavily pretreated patient with gastric cancer and liver metastases [6]. In a recent phase II trial conducted in Japan, everolimus 10 mg/day was administered to 53 patients with previously treated metastatic gastric cancer [19]. Although no complete or partial responses were documented, 45% of patients had a decrease in tumor size from baseline by independent radiologic review. In another Korean phase II trial of 54 patients with chemotherapy-refractory advanced gastric cancer treated with everolimus 10 mg/day, two (4%) patients achieved confirmed partial responses [6].

The GRANITE-1 (gastric antitumor trial with everolimus-1) phase III trial compared everolimus/supportive care with placebo/best supportive care in previously treated patients with advanced gastric cancer. In this trial, everolimus has been found to be active, significantly improving PFS (hazard ratio, 0.66; 95% CI, 0.56–0.78; *P* < 0.001), but OS was not significantly prolonged (hazard ratio, 0.90; 95% CI, 0.75–1.08; *P* = 0.1244) [23].

Taken together, the data show that everolimus has some activity in gastric cancer but objective responses have been rare. This raises the questions of how many gastric cancer patients really harbor inappropriate mTOR activation and of whether the administration of everolimus to unselected patient populations is meaningful. Patient selection based on molecular events known to be associated with mTOR activation such as the overexpression of *PI3K*/*Akt* and the growth factor receptors human epidermal growth factor receptor 2 (HER2) and insulin-like growth factor receptor (IGFR) as well as mutations in *PI3K* and mutations/amplifications of *Akt* or downregulation of PTEN may represent an appropriate way to identify populations that are more likely to show significant benefit from mTOR inhibitors. Unfortunately, little was known about the molecular profiles of patients who responded to everolimus in initial trials. This has led us to incorporate a biomarker analysis in our study, which found genetic alterations related to *PTEN* in 12.5% (2/16) of patients and related to *AKT1* in one patient. Alterations of *PTEN* by inactivating mutations and/or chromosomal deletions have been described in many different tumor types including gastric cancer [24]. In our study, we found one mutation in Exon 2 at Codon 36 (p.G36E), which is published as a hot spot region and might have a crucial role in the carcinogenesis or progression of gastric cancer [7]. The other alterations including SNP rs1903858 (*PTEN*) and SNP rs3730358 (*AKT1*) were intronic variants that did not seem to have any functional consequences, as they did not affect splice junctions. In addition, we detected an overexpression of HER2 in 20% (3/15) of patients. The responders did not harbor any remarkable genetic alterations, but the number of patients in this phase I study and the number of factors evaluated and found were too limited to enable the discovery of relevant biomarkers in our study. It is very important that future studies identify potential markers of response to everolimus and validate their role. An ongoing phase III trial (*n* = 480) of our group (RADPAC trial) evaluates paclitaxel monotherapy with or without everolimus in the second- or third-line setting (NCT01248403). The study administers everolimus to an unselected patient population, but is accompanied by a comprehensive exploratory biomarker research programs including next-generation sequencing to identify genetic variants with potential link to everolimus activity. We used MMC in the present study, because it was the only approved drug for gastric cancer in the second-line setting and could build an acceptable comparator for a further phase III trial. However, the decision to combine everolimus with paclitaxel and not with MMC in the RADPAC trial mentioned above study was met after comprehensive discussions mainly based on the encouraging results of everolimus/paclitaxel combination achieved in larger patient populations with breast cancer.

In conclusion, the results of our phase I study suggest that oral everolimus can be safely administered at 10 mg/day in combination with the cytotoxic drug MMC at 5 mg/m^2^ every 3 weeks in previously treated patients with gastric cancer and adenocarcinoma of the EGJ. Encouraging signs of antitumor activity were observed, indicating that the combination of everolimus and cytotoxic drugs for gastric cancer deserves further evaluation.
